# Generation of High Affinity Anti-Peptide Polyclonal Antibodies Recognizing Goat α_s1_-Casein

**DOI:** 10.3390/molecules25112622

**Published:** 2020-06-05

**Authors:** Aliah Zannierah Mohsin, Rashidah Sukor, Jinap Selamat, Anis Shobirin Meor Hussin, Intan Hakimah Ismail, Nuzul Noorahya Jambari, Farina Mustaffa-Kamal

**Affiliations:** 1 Institute of Tropical Agriculture and Food Security, Universiti Putra Malaysia, Serdang 43400, Malaysia; aliahmohsin89@gmail.com (A.Z.M.); sjinap@gmail.com (J.S.); noorahya@upm.edu.my (N.N.J.); 2 Faculty of Food Science and Technology, Universiti Putra Malaysia, Serdang 43400, Malaysia; shobirin@upm.edu.my; 3 Faculty of Medicine, Universiti Putra Malaysia, Serdang 43400, Malaysia; intanhakimah@upm.edu.my; 4 Faculty of Veterinary Medicine, Universiti Putra Malaysia, Serdang 43400, Malaysia; farina@upm.edu.my

**Keywords:** IgG purification, goat’s milk allergy, α_s1_-casein, anti-peptide polyclonal antibody, immunogenic peptides

## Abstract

The chemical, technological and allergy properties of goat’s milk are significantly affected by the level of α_s1_-casein. Detection and quantification of α_s1_-casein requires high-specificity methods to overcome high-sequence similarity between this protein and others in the casein family. Unavailability of antibodies with high affinity and specificity towards goat α_s1_-casein hinders the development of immuno-based analytical methods such as enzyme-linked immunosorbent assay (ELISA) and biosensors. Here, we report the generation of polyclonal antibodies (or immunoglobulins, IgGs) raised towards goat α_s1_-casein N- (Nter) and C-terminal (Cter) peptide sequences. The Nter and Cter peptides of goat α_s1_-casein were immunized in rabbits for the generation of antisera, which were purified using protein G affinity chromatography. The binding affinity of the antisera and purified IgGs were tested and compared using indirect ELISA, where peptide-BSA conjugates and goat α_s1_-casein were used as the coating antigens. The Nter antiserum displayed higher titer than Cter antiserum, at 1/64,000 and 1/32,000 dilutions, respectively. The purification step further yielded 0.5 mg/mL of purified IgGs from 3 mL of antisera. The purified Nter IgG showed a significantly (*p* < 0.05) higher binding affinity towards peptide-BSA and goat α_s1_-casein, with lower K_d_ value at 5.063 × 10^−3^ μM compared to 9.046 × 10^−3^ μM for the Cter IgG. A cross-reactivity test showed that there was no binding in neither Nter nor Cter IgGs towards protein extracts from the milk of cow, buffalo, horse and camel. High-quality antibodies generated will allow further development of immuno-based analytical methods and future in vitro studies to be conducted on goat α_s1_-casein.

## 1. Introduction

Goat α_s1_-casein is a phosphorylated protein and has been identified as a major allergen in goat’s milk. The α_s1_-casein production levels in different goats may vary greatly due to the high polymorphism of the CSN1N1 gene that encodes the α_s1_-casein. Various levels of α_s1_-casein synthesis in goat’s milk have been described, ranging between 0 to 3.5 g/L for null alleles (N, O) and strong alleles (A, B, C, H, L, M), respectively [1]. The genetic polymorphism of the goat α_s1_-casein has been shown to substantially affect the chemical composition and technological properties of the milk [2,3,4], including the allergic reactions caused by α_s1_-casein [5,6]. Determining the level of goat α_s1_-casein in milk samples is therefore important for the safety and quality assessment of the goat’s milk or goat’s milk-based products. The detection and quantification of α_SI_-caseins require analytical methods with high specificity, high sensitivity, rapidity, robustness, reliability and cost-effectiveness. Immuno-based analytical methods such as enzyme-linked immunosorbent assay (ELISA) and biosensors are ideal platforms for this purpose, but they demand high quality antibodies as their biorecognition molecules. However, unavailability of the antibodies that specifically recognize goat α_s1_-casein has hampered the development of these methods.

Antibodies or immunoglobulin (IgGs) are heterodimer proteins produced by the B lymphocytes of the immune system of vertebrates. They play important roles in binding to the specific target antigens and in eliciting immune responses against that target antigen. The association between the antibody and antigen involves a myriad of noncovalent interactions between the epitope and the paratope. This association has exquisite specificity and a high affinity, making the antibody an enormously valuable tool in many biomedical research fields, diagnostics and therapy [7].

The traditional way of producing antibodies is by immunizing the host animal using whole proteins that are purified from a biological source or produced via a recombinant technology, such as immunogens. This method may be favored, as the antibodies generated could recognize multiple epitopes on the protein and thus maximize the affinity binding between the antibodies and the protein. Nevertheless, the quality of the antibodies produced may not be applicable, due to qualities such as low specificity and high cross-reactivity with protein families that share high sequence similarities. For example, casein in goat’s milk, consisting of α_s1_-, α_s2_-, β- and κ-casein share 95% homology of amino acid identities, as well as casein from the milk of other ruminants. Instead of using the whole protein as an immunogen, the production of antibodies using synthetic peptides has increased in the specificity of antibodies to the target antigens [8,9,10,11,12]. Additionally, this method provides an alternative to raise antibodies for a specific protein isoform, phosphorylated or glycosylated proteins, and proteins that are not easily purified, such as large membrane proteins [12].

Antibodies are usually isolated from plasma, serum, ascites fluid, cell culture medium, egg yolk, plant extracts or bacterial and yeast cultures. These sources contain a large number of extraneous proteins that can interfere with the detection system, potentially causing a high background or low binding of the antibody [13]. Hence, efficient purification of the antibody is required. Antibody purification can be achieved through a range of methodologies, including precipitation, electrophoretic separations, filtration, and liquid chromatography [13]. Among them, affinity chromatography was proven to be the most efficient and widely employed [14,15].

This study aims to generate high affinity anti-peptide polyclonal antibodies (IgGs) towards native goat α_s1_-casein. The produced IgGs have high potential and are applicable in the development of immuno-based assays for the detection and quantification of native goat α_s1_-casein, such as ELISA and biosensors. Such antibodies will be a valuable addition to its availability and important for experimental applications on goat α_s1_-casein.

## 2. Results and Discussion

### 2.1. Humoral Response

**Figure 1:**
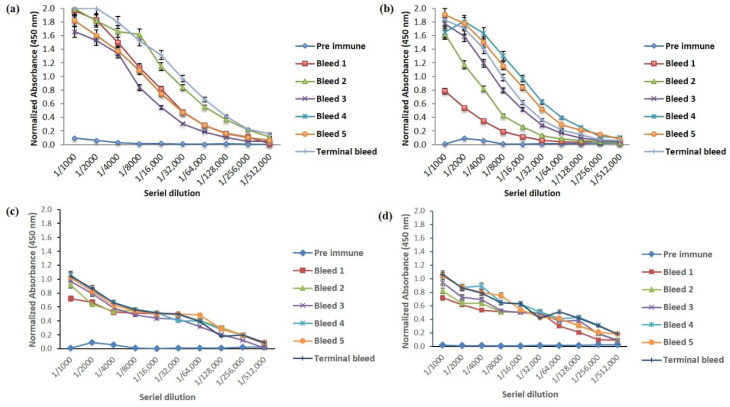
Titers of rabbit antiserum immunized with peptide-KLH; (**a**) rabbit 1, Nter-KLH; (**b**) rabbit 2, Nter-KLH; (**c**) rabbit 1, Cter-KLH; (**d**) rabbit 2, Cter-KLH.

Antisera titer is defined as antisera dilution, resulting in an uninhibited assay signal three times that of the background signal; in this case, preimmune sera is investigated under the given assay conditions. It was determined through an end-point titration ELISA. Most antisera showed fairly high titers for their homologous antigens; typical reciprocal titers were in the order of 10^3^ to 10^5^ [16]. Rabbit 1 (terminal bleed) immunized with Nter peptide-KLH showed a higher titer at 1/128,000 than rabbit 1 (terminal bleed) immunized with Cter peptide-KLH, which had a lower response towards the immunogen with a titer at 1/64,000 dilution of antisera. As shown in Figure 1, the titer of antisera for both rabbits were considered high when compared to previous studies [17,18].

The number of amino acids between the peptides may have contributed to the difference in the response towards the immunogen, where Nter had 19 amino acids (2180.49 Da), while Cter had 16 amino acids (1719.80 Da). The number of peptides from 10 to 20 amino acids are considered to be optimal immunogens [19,20]. Short peptides (less than seven amino acids) may be insufficiently sized to function as epitopes due to the smaller molecular size. In contrast, larger peptides may adopt their own specific conformation, which may not be reflected in the conformation of the sequence within the intact protein [19,20]. Even though both peptides had an optimal number of amino acids as immunogens, the immunization of a larger peptide antigen from a protein may statistically increase the chances of obtaining antibodies that recognize the target protein [20].

Apart from the molecular weight, both peptides also had different hydrophilicity, which was strongly influenced by the amino acid composition [19,20,21,22]. Nter had better hydrophilicity than Cter because it was capped with hydrophilic amino acids (Arg and Glu) at both ends of the peptide. Furthermore, the ratio of hydrophilic residues to the total number of residues for peptide Nter (44%) was also higher than peptide Cter (33%). Additionally, both peptides were in accordance to [22], who proposed that peptide antigens should contain at least 30% of hydrophilic amino acids. Better hydrophilicity increased the solubility of the Nter peptide, subsequently increasing the probability of the peptides to be available at the surface of the protein for antibody binding.

One important criteria when raising anti-peptide antibodies is to find a suitable peptide in a database of experimental epitopes [20]. B-cell epitopes, which are protein antigens that are recognized by the B-cells, are classified as either continuous (linear) or discontinuous (conformational) epitopes. Many B-cell epitope databases or prediction servers can assist in maximizing the success rate of producing antibodies of a high quality; however, most of them can only predict a continuous epitope. Kapila et al. [23] listed the B cell and T-cell epitopes for α_s1_-casein from cow and buffalo, but none for goat α_s1_-casein, due to unavailable literature. Hence, a B cell epitope prediction server, such as BepiPred B-cell epitope prediction server (BepiPred, https://services.healthtech.dtu.dk/service.php?BepiPred-2.0) [20]. Table 1 shows the B cell epitope probability from BepiPred. The probability of Nter for B cell epitope was higher (0.424–0.653) than Cter (0.303–0.610). A higher peptides score indicates a higher probability of it being an epitope. 

The conjugation of the peptide to the carrier protein is one of the critical factors in antibody production. Since both peptides are hapten, conjugation to a carrier protein is necessary to induce a sufficient immune response [19,20,21]. In this study, KLH was chosen because it is large in size (4.5 × 10^5^–1.3 × 10^7^ Da) and has numerous epitopes that helps to illicit T helper- and B-cell responses in animals. It also contains an abundance of lysine residues for peptide coupling, which allows for a higher peptide–carrier protein ratio, thus increasing the likelihood of generating peptide-specific antibodies. In addition, KLH is phylogenetically distant from mammalian proteins as it is derived from a gastropod limpet. This reduces false positives in immune-based research techniques in mammalian model organisms [24]. It is critical that the molar ratio of peptide to carrier protein to be high; one mole of peptide per 50 amino acids of carrier is a reasonable coupling ratio. In our case, the peptide to KLH molar ratio used was 2:1, and was appropriate according to other references [12,21,22,25]. 

Polyclonal antibodies were raised in this study, as opposed to monoclonal antibodies (mAb), which may have offered higher specificity. This set-back was compensated for by raising the antibodies with a synthesized peptide derived from a particular region of the α_s1_-casein amino acid sequence. The selection of pAbs over mAbs was mainly due to the relatively easier production and lesser cost, and because pAbs can be obtained in a reasonably shorter period. Peptide antigens are also more often used to generate pAbs—mainly comprised of the IgG subclass in either goats or rabbits, which target unique epitopes [20]. The quality of the antibodies varies among animals; therefore, more than one rabbit is required with a detailed selection of rabbits. A large pool of antibodies was also collected to support the long-term commercial production of the uniform product. 

### 2.2. Purification of Polyclonal Antibody (pAb) Using Affinity Chromatography

**Figure 2:**
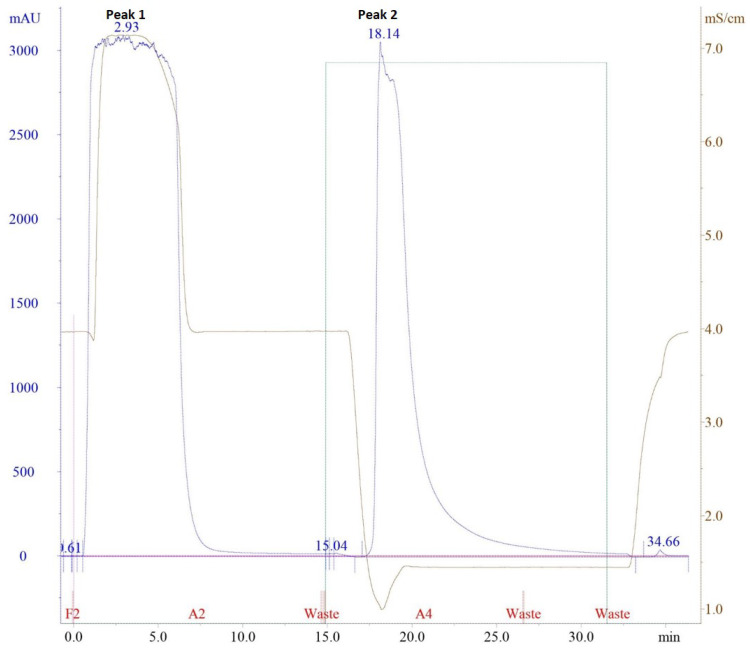
Chromatographic profile by immunoglobulins (IgG) obtained by 20 mM sodium phosphate as the binding buffer (Peak 1) and 0.1 M glycine-HCl as the elution buffer (Peak 2) identified at 280 nm. Blue line: absorbance (mAU), brown line: conductivity (mS/cm), and green line: concentration of solvent B (%).

The purification of antibodies (IgGs) by protein G, immobilized on an affinity matrix took advantage of its domain structure, which targets the constant part of the antibodies—the Fc region. The process could be monitored based on the conductivity of the solvent (mS/cm), absorbance (mAU) and concentration of the elution buffer (%), as shown in Figure 2. It required 20 mM sodium phosphate (pH 7) as the binding buffer, since the IgG from most species and subclasses bind to protein G close to the physiological pH and ionic strength [26]. When the serum sample was introduced, the conductivity of the binding buffer increased from the second to seventh minute. This was due to the increased salt concentration as the serum sample was also diluted in the binding buffer. The binding peak with a five-column volume (CV) could be identified as Peak 1 at 280 nm. To elute the IgG, 0.1 M glycine-HCl (pH 2.7) was used as the elution buffer. The positive charge of HCl interfered with the IgG-protein G association and they were readily dissociated at a low-pH elution buffer. In Figure 2, when 100% elution buffer was used, the conductivity decreased at minute 17 because the salt concentration reduced to pH 2.7. The elution peak was identified as the Peak 2. To preserve the activity of acid-labile IgG, 1 M of Tris-HCl was added into the collection tubes. The yield of IgGs obtained from 3 mL of rabbit antisera was 0.5 mg/mL. 

**Figure 3:**
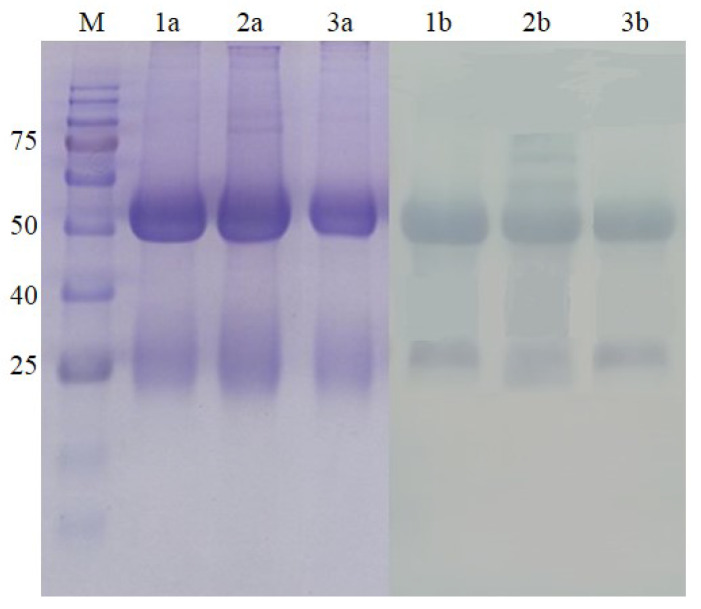
SDS-PAGE (a) and Western blot (b) shows two IgG bands, heavy chain at 50 kDa and light chain at 25 kDa using 12% separating gel. Lane 1: standard IgG from rabbit, Lane 2: purified Nter IgG. Lane 3: purified Cter IgG. Each lane was loaded with 20 μg/mL of protein.

The purity of both Nter and Cter IgGs was assessed based on Sodium dodecyl sulphate-polyacrylamide gel electrophoresis (SDS-PAGE). The basic structure of an antibody is composed of two identical heavy (H) chains (molecular weight 50 kDa) and two identical light (L) chains (molecular weight 25 kDa) that are linked together by disulfide bonds. Reducing the buffer containing 2% (*v*:*v*) of 2-mercaptoethanol caused a break in the disulfide bonds. As shown in Figure 3, two bands were observed at 50 kDa and 25 kDa, which depicted the heavy and light chain of the IgG. The light chain appeared as a diffused band and could be related to many factors, such as the sample being overloaded or degraded or the sample being in a high-salt buffer [27]. Overall, the purification of pAbs by affinity chromatography resulted in a total IgG that was highly pure, as similarly shown in Majidi et al. [17].

### 2.3. Binding of Purified IgGs Towards Peptides-BSA and Goat α_s1_-Casein Using Indirect ELISA

**Figure 4:**
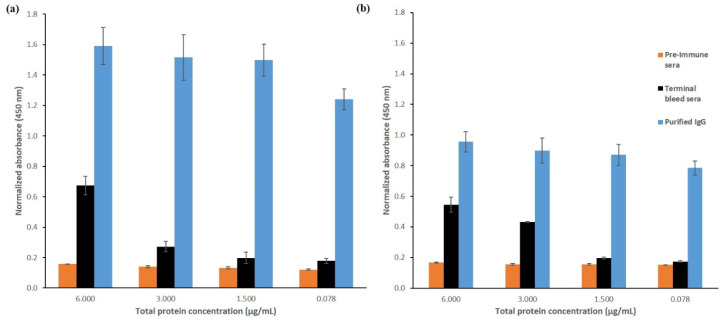
Comparison between binding of purified IgG, terminal bleed and preimmune sera from rabbits immunized with (**a**) Nter-KLH, (**b**) Cter-KLH.

The purification of antibodies could enhance their binding by removing nonspecific molecules that might interfere with the interaction of antibodies towards the target antigen—in this case peptides. Figure 4 shows the binding of Nter and Cter IgGs, in comparison to the binding of terminal bleed and preimmune sera, towards peptide-BSA. At a comparable protein concentration, terminal bleed antisera showed significantly lower binding towards peptide-BSA than the purified IgGs. Considering other proteins such as albumin coexisted in the antisera, the binding of these proteins towards peptide-BSA were not specific, as shown in low signal in preimmune sera. Normal rabbit serum contains about 75% of IgG [28]. Purification of the immunoglobulin using affinity chromatography has significantly improved the binding of Nter and Cter IgGs towards peptide-BSA.

**Figure 5:**
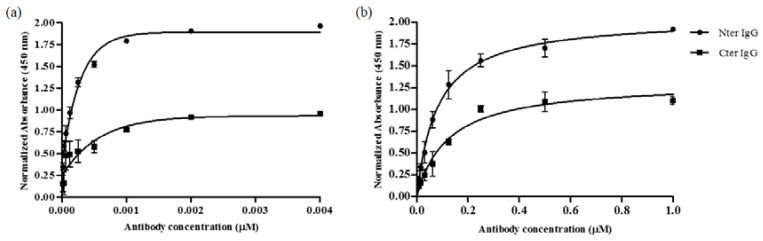
The comparison between the binding of Nter IgG and Cter IgG against (**a**) peptide-BSA conjugates and (**b**) goat α_s1_-casein using indirect ELISA.

Figure 5 shows a comparison between the binding of purified polyclonal IgG of Nter and Cter peptides towards (a) peptides-BSA and (b) goat α_s1_-casein, respectively. Both IgGs were from the terminal bleed (day 161). At the highest concentration of IgG (0.004 μM for peptide-BSA and 1.0 μM for goat α_s1_-casein), Nter IgG showed significantly higher binding towards both peptide-BSA and goat α_s1_-casein than Cter IgG. The result was in parallel to the findings from the humoral response test (Section 2.1). 

K_d_ is the dissociation constant between the antibody and its antigen. A lower K_d_ value signifies a higher affinity of an antibody. For polyclonal antibodies, the K_d_ value is an average of the total IgGs in the polyclonal mixture and not limited to the IgG of the target antigen; any one antibody in the mixture could be much stronger or weaker than the average. The affinity of purified polyclonal IgGs of Nter and Cter towards peptides-BSA conjugates showed that K_d_ of Nter IgG (1.09 × 10^−4^ μM) was significantly (*p* < 0.05) lower than the K_d_ of Cter IgG (1.12 × 10^−4^ μM). A similar trend was observed when goat α_s1_-casein was used as the coating antigen, where the K_d_ of Nter IgG (5.063 × 10^−3^ μM) was significantly (*p* < 0.05) lower than the K_d_ of Cter IgG (K_d_ 9.046 × 10^−3^ μM). Nevertheless, the K_d_ value for both IgGs towards goat α_s1_-casein was larger than the K_d_ for peptides-BSA, which indicates that both IgGs had lower affinity towards native goat α_s1_-casein than the peptides-BSA. 

Cases where an antibody has a high titer towards peptide than the native protein could be explained by several possibilities. The peptide sequence may correspond to a nonexposed region of the native protein. Additionally, the conformation of protein in the peptide region may differ from the peptide, which subsequently causes the antibody to have difficulty in recognizing the native protein [20]. Antibodies only bind to epitopes found on the surface of the proteins and tend to bind with higher affinities when those epitopes are flexible enough to move into accessible positions [20]. The position of the epitope in peptides-BSA might be more accessible than the epitope on the structure of the native goat α_s1_-casein. The limitation of this study is that we could not determine the orientation of the peptides-BSA and goat α_s1_-casein as the coating antigens. The epitope might be hindered by the macro structure of BSA or goat α_s1_-casein. Regardless of that, the results showed that both IgGs had good affinities towards the native goat α_s1_-casein and the ability of anti-peptide IgGs to recognize the whole native protein of the goat α_s1_-casein was proven. 

### 2.4. Cross Reactivity

**Figure 6:**
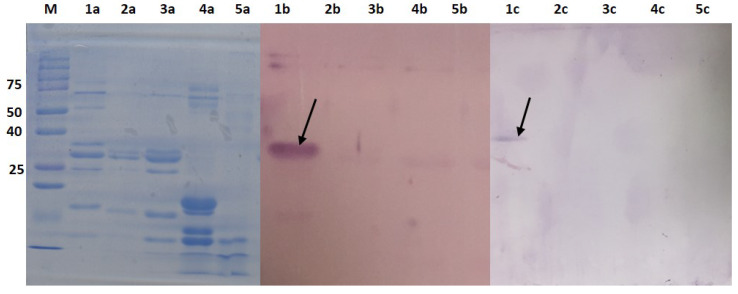
Western blot shows for total protein extract from 1: goat’s milk; 2: cow’s milk, 3: buffalo’s milk; 4: horse’s milk; 5: camel’s milk. a: SDS-PAGE; b: immunoblot for Nter IgG, c: immunoblot for Cter IgG. Each lane was loaded with 20 μg/mL of protein. Nter and Cter IgGs were loaded at 8 and 16 μg/mL, respectively.

Western blot was conducted using protein extracts from milk of goat, cow, buffalo, horse and camel to confirm the specificity of the antibodies towards α_S1_-casein found in goat’s milk. Based on Figure 6, lane 1 (goat’s milk) showed a single band at 25–37 kDa which represents the α_S1_-caseins. Other lanes did not show any band which depicted no binding between the antibodies and the milk protein extract from other mammals. Thus, the result provides confirmation of the specificity of both the Nter and Cter IgGs towards goat α_S1_-casein. 

Protein phosphorylation is the addition of the phosphate group to specific amino acids such as serine, threonine or tyrosine residues on proteins. Goat α_s1_-casein is a protein with several phosphorylation sites. It is important to ensure that the antibodies produced has limited binding to the phosphorylated sites of the protein. Available database on phosphorylated sites in protein could be found at http://www.phosphosite.org/homeAction.do, unfortunately no database was available for goat α_s1_-casein. Therefore, the preliminary prediction of goat α_s1_-casein phosphorylation is the prediction servers such as Netphos (http://www.cbs.dtu.dk/services/NetPhos/). Based on the prediction, goat α_s1_-casein has numerous numbers of phosphorylated sites, as shown in Figure S1. As the antibodies are raised against peptides, only phosphorylated sites found in the peptide is of concern. Both peptides showed one site of phosphorylation which obtained high score (~1); Nter: HRGLSPEVP (score: 0.988); Cter: GSENSGKTT (score: 0.970). A high score indicates the high likelihood of phosphorylation sites, however, a small number of phosphorylation sites reduces the chances for the antibodies to recognize the phosphorylated sites compared to nonphosphorylated sites. The limitation of this study is that the confirmation test to discriminate between phosphorylated and nonphosphorylated sites of the peptide was not carried out. Thus, the antibodies “may” cross-react with corresponding phosphorylated goat α_s1_-casein.

## 3. Materials and Methods

### 3.1. Materials

Peptide Nter and Cter were synthesized and conjugated by SBS Genetech Co., Ltd. (Beijing, China). Four New Zealand White (NZW) female rabbits (12 to 14 weeks) were purchased from A Sapphire (Selangor, Malaysia). Skimmed milk was purchased from Becton Dickinson (Franklin Lakes, NJ, USA). Titermax Gold Adjuvant, Freund’s Incomplete Adjuvant, sodium chloride (NaCl), sodium hydroxide (NaOH), potassium chloride (KCl), sodium phosphate dibasic (Na_2_HPO_4_.2H_2_0), potassium dihydrogen phosphate (KH_2_PO_4_)_,_ sodium phosphate (NaPO_4)_ and 2-mercaptoethanol were purchased from Sigma-Aldrich (St. Louis, MO, USA). EMLA^®^ cream (AstraZeneca, Cambridge, UK) and pentobarbital/Nembutal^®^ (Abbott Laboratories, Chicago, IL, USA) were provided by attending a veterinarian. Nunc Maxisorp 96-well microtiter plates, 12–14 kDa MWCO dialysis tubing (Spectrum Labs), and 1-Step Ultra TMB-ELISA substrate were obtained from Thermo Fisher Scientific (Waltham, MA, USA). HiTrap Protein G HP column (1 mL) was purchased from GE Healthcare (Uppsala, Sweden). Tween 20, sulphuric acid (H_2_SO_4_), hydrochloric acid (HCl), acetic acid, TEMED (N,N,N′,N′-tetramethylethylenediamine), methanol, NBT-BCIP and Immobilon^®^-P PVDF membrane were obtained from Merck & Co. (Kenilworth, NJ, USA). Peroxidase AffiniPure goat anti-rabbit IgG (H + L) and Alkaline phosphatase AffiniPure goat anti-rabbit IgG (H + L) were obtained from Jackson ImmunoResearch Laboratories (West Grove, PA, USA). Sodium dodecyl sulfate, glycine, Tris and Coomassie Blue G 250 were purchased from Bio-Rad Laboratories (Hercules, CA, USA). Deionized water (18.3 MΩ.cm) was obtained from ELGA LabWater (High Wycombe, UK).

### 3.2. Synthesis of Peptide

Two peptides derived from amino acid sequences of goat α_s1_-casein were synthesized. Peptide one was identified as Nter, which consisted of 18 amino acids (RPKHPINHRGLSPEVPNE, fragment 1–18 of goat α_S1_–casein, molecular weight: 2077.04 kDa, purity: 95.39%). Peptide two was identified as Cter, consisting of 15 amino acids (PIGSENSGKTTMPLW, fragment 185–199 of goat α_S1_–casein, molecular weight: 1617.67 kDa, purity: 85.25%). Both peptides were conjugated to keyhole limpet hemocyanin (KLH) for animal immunization and to bovine serum albumin (BSA) for coating antigens in ELISA. 

### 3.3. Immunization of Animals

The animal study conducted was approved by Institutional Animal Care and Use Committee (IACUC), Universiti Putra Malaysia (AUP-R042/2016). Four New Zealand White (NZW) rabbits (12 to 14 weeks) were used for the production of polyclonal antibodies. Upon arrival, the primary weight of each rabbit was recorded and primed in the Animal House, Faculty of Veterinary Medicine, Universiti Putra Malaysia for seven days. Rabbit immunization was carried out according to antibody production guideline from Canadian Council on Animal Care and He et al. (2017) with some modifications [29,30]. For primary immunization (injection 1), 200 μg of peptide-KLH was dissolved in 1 mL of saline and mixed with Titermax Gold Adjuvant at 1:1 (*v:v*) ratio. For subsequent injections or boosters (injection 2 until final injection), 100 μg of peptide-KLH was dissolved in 1 mL saline and mixed with Incomplete Freud Adjuvant at similar ratio. Injections of 0.25 mL of emulsified immunogen per site were performed at four separated subcutaneous sites involving two inguinal and axillary sites. Bloods (5 mL) were collected from each rabbit through marginal ear vein, seven days after each injection. Before blood withdrawal, 5% EMLA cream (2.5% lidocaine, 2.5% prilocaine) was applied topically on the area. The rabbits were euthanized by over dosage of pentobarbital (100 mg/kg) under anesthesia via intraperitoneal injection. Terminal bleed was obtained after 161 days through cardiac puncture. Bloods were allowed to clot overnight at 4 °C and centrifuged at 1800× *g* for 10 min. The antisera collected were stored at −20 °C until further analysis. 

### 3.4. Humoral Response ELISA

Nunc Maxisorp high binding 96-well ELISA plates were coated overnight at 4 °C with 100 µL of 10 μg/mL of peptide Nter-BSA or peptide Cter-BSA conjugates in phosphate buffer saline (PBS). The next day, the plates were blocked using 5% skimmed milk in PBS and incubated for 2 h at room temperature. The plates were washed thrice with PBST (0.5% Tween 20 in PBS) and further incubated with antisera (100 µL) at starting dilution of 1:1000 (*v*:*v*) for 1 h at room temperature. Washing steps were repeated (5×) and the plates were incubated with 100 µL of Peroxidase AffiniPure goat anti-rabbit IgG (1:10,000; *v*:*v*) at similar condition. The plates were then washed (3×) and ELISA reaction was initiated by addition of 1-Step Ultra TMB-ELISA substrate (100 µL). The reaction was quenched by the addition of 0.1 M H_2_SO_4_ (100 µL). The resulting absorbance was measured with a Multiscan RC Microplate reader (Thermo Fisher Scientific, MA, USA) at 450 nm.

### 3.5. Affinity Chromatography

The antisera from rabbit 1 immunized with Nter- and Cter were subjected to affinity chromatography using HiTrap Protein G HP column according to manufacturer’s instruction. The purification employed 20 mM sodium phosphate (pH 7) as the binding buffer, and 0.1 M glycine-HCl (pH 2.7) as the elution buffer. About 3 mL of antisera were diluted in 4 mL of binding buffer, filtered using 0.45 μm membrane filter, and subjected to Fast Protein Liquid Chromatography system (GE Healthcare, Uppsala, Sweden) using a 10 mL sample loop at 1 mL/min. The elution peak was collected in a 15 mL tube containing 100 μL of 1 M Tris-HCl (pH 9). The eluted IgGs were then dialyzed against PBS (12–14 kDa MWCO) and lyophilized. The lyophilized antibody was weighed and kept at −20 °C until further analysis. 

### 3.6. Milk Samples Preparation for Western Blot

Milk samples (goat, cow, buffalo and horse) were collected from commercial farms in Malaysia while commercial camel milk was purchased from the market. Milk samples were aliquoted in 2 mL tubes and centrifuged at 1800× *g* for 30 min at 4 °C. The skimmed milk between the top fat layer and bottom pelleted cells was transferred into new 2-mL tubes and immediately underwent protein extraction. The total milk protein extraction method was adapted from Vincent et al. [31]. 

### 3.7. SDS-PAGE and Western Blot Analysis

The IgG fractions before lyophilization were assessed by SDS-PAGE using 12% polyacrylamide gel as previously described [27]. For reducing conditions, 2% (*v*:*v*) of 2-mercaptoethanol was added to the sample buffer and boiled for 5 min. The sample was electrophoresed for 60 min at a constant voltage of 110 V. The gels were either stained overnight using Coomassie Blue G 250 in methanol:acetic acid:water (40:10:50) or electroblotted onto PVDF membrane using Trans Blot Semi-dry Transfer Cell (Bio-Rad Laboratories, CA, USA). Briefly, the membrane was blocked with 5% skimmed milk for 2 h. After 3 washes with PBST, the membrane was incubated with 1 in 15,000 dilution (0.04 μg/mL) of alkaline phosphatase AffiniPure goat anti-rabbit IgG (H + L) for 1 h. The immunoblot was developed using NBT-BCIP substrate. The purity of the IgG fraction was determined based on the number of bands visible on the gel. 

### 3.8. Purification of α_s1_-Casein from Goat’s Milk 

**Figure 7:**
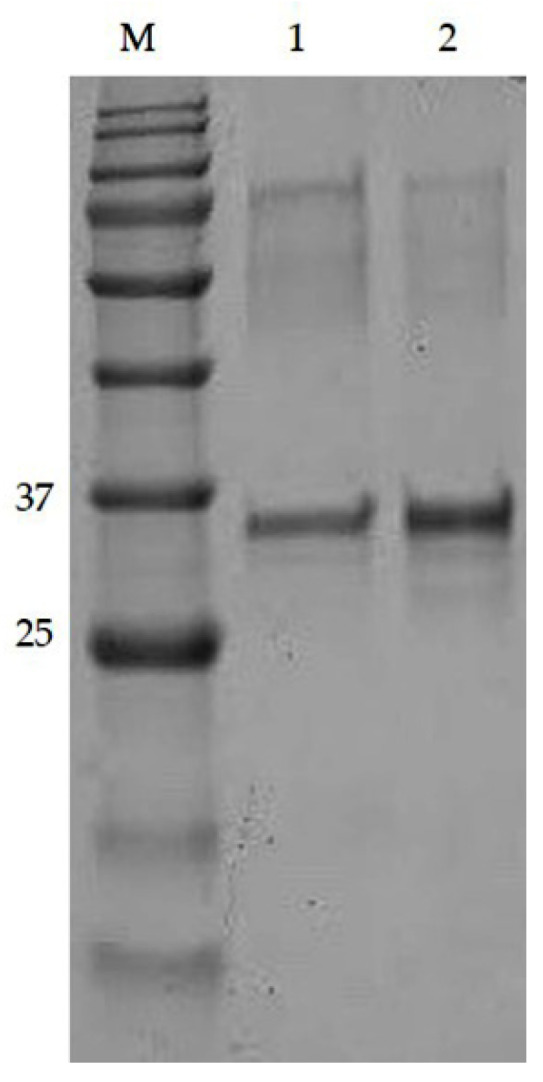
SDS-PAGE showed a single band of α_s1_-casein at ~30 kDa. The lane was loaded with different protein concentrations: Lane 1:10 μg/mL, Lane 2:20 μg/mL, M: Protein marker.

The protein standard of goat α_s1_-casein was not commercially available. Therefore, the protein was purified according to previous study [32]. Assessment of the α_s1_-casein by SDS-PAGE showed the protein to be a single band. Identification of the protein was confirmed by molecular weight on SDS-PAGE as shown in Figure 7, and LC-MS/MS, as shown in Figure S2 and Table S1.

### 3.9. Indirect ELISA

Indirect ELISA to examine the affinity of purified IgGs towards peptides-BSA was carried out as described in Section 3.4. Polyclonal IgGs previously obtained from affinity purification were used as primary antibody. 

### 3.10. Data Analyses

All data were done in triplicates (*n =* 3). Absorbance without addition of antisera or IgGs were treated as control (A_o_). Data was analyzed and plotted using Microsoft Excel (Microsoft Office ver. 2013, Microsoft Corporation, Redmond, WA, USA). GraphPad Prism 5 (San Diego, CA, USA) was applied to analyze nonlinear regression model; Four parameter log graph (4PL) [Y = Bottom + (Top − Bottom) / (1 + 10^((LogEC50 − X) × HillSlope))] to represent standard curve of normalized absorbance versus serial dilution of antisera and purified IgGs, and one-site binding model [Y = Bmax × X/(K_d_ + X)] to determine the affinity constant (K_d_) of purified IgGs.

## 4. Conclusions

The usual technique to determine if a selected peptide antigen can produce good antibodies to recognize the native protein is through immunization of the peptide into animals. However, since most peptides have smaller molecular sizes, they may not be immunogenic. In this study, anti-peptide polyclonal antibodies were designed, based on the N-terminal and C-terminal amino acid sequence of goat α_s1_-casein. Based on the results, Nter and Cter IgGs could bind to the peptide epitopes on goat α_s1_-casein with K_d_ of 5.063 × 10^−3^ µM and K_d_ 9.046 × 10^−3^ µM, respectively. Both IgGs also showed high specificity towards goat α_s1_-casein, showing significantly (*p* < 0.05) lower binding towards cow α-casein. The produced IgGs have high potency in order to be applied in the development of analytical methods for the detection and quantification of native goat α_s1_-casein such as ELISA. The developed ELISA with high specificity and sensitivity towards goat α_s1_-casein will be used for the assessment of this protein in milk and milk products. It will substantially benefit several parties which include goat breeders in the breed selection process, goat’s milk product manufacturers for cheese making, health practitioners, as well as consumers on selection of hypoallergenic products from goat’s milk. Additionally, the generated antibodies which can be further explored for research purposes can be made available.

## Figures and Tables

**Table 1:** Summary results from BepiPred for each amino acid of Nter and Cter peptides, which include helix, sheet, coil and epitope (B-cell) probability.

Position	Amino Acid	Exposed/Buried	Relative Surface Accessibility	Helix Probability	Sheet Probability	Coil Probability	Epitope Probability
1	R	B	0.184	0.176	0.004	0.82	0.424
2	P	B	0.234	0.058	0.017	0.925	0.464
3	K	E	0.515	0.018	0.019	0.964	0.508
4	H	B	0.151	0.018	0.019	0.964	0.540
5	P	E	0.315	0.058	0.017	0.925	0.594
6	I	B	0.16	0.053	0.043	0.903	0.621
7	N	E	0.473	0.053	0.043	0.903	0.619
8	H	B	0.262	0.058	0.017	0.925	0.609
9	R	E	0.458	0.113	0.043	0.844	0.620
10	G	E	0.604	0.113	0.087	0.8	0.627
11	L	B	0.271	0.113	0.087	0.8	0.643
12	S	B	0.302	0.053	0.043	0.903	0.650
13	P	E	0.571	0.053	0.043	0.903	0.654
14	E	E	0.571	0.053	0.043	0.903	0.627
15	V	E	0.469	0.184	0.043	0.773	0.623
16	P	E	0.476	0.184	0.043	0.773	0.632
17	N	E	0.517	0.184	0.043	0.773	0.634
18	E	E	0.517	0.191	0.086	0.723	0.639
185	P	B	0.205	0.018	0.047	0.935	0.572
186	I	B	0.275	0.018	0.088	0.893	0.576
187	G	B	0.195	0.018	0.088	0.893	0.594
188	S	B	0.278	0.018	0.088	0.893	0.594
189	E	E	0.622	0.053	0.043	0.903	0.596
190	N	E	0.525	0.053	0.043	0.903	0.596
191	S	B	0.298	0.053	0.043	0.903	0.605
192	G	E	0.599	0.053	0.043	0.903	0.604
193	K	E	0.517	0.052	0.084	0.864	0.611
194	T	B	0.239	0.02	0.205	0.775	0.603
195	T	E	0.383	0.02	0.205	0.775	0.588
196	M	B	0.247	0.018	0.141	0.84	0.514
197	P	E	0.376	0.018	0.088	0.893	0.439
198	L	E	0.581	0.018	0.088	0.893	0.366
199	W	E	0.497	0.003	0.003	0.994	0.303

## Supplementary Materials

The following are available online, Figure S1: The prediction results for phosphorylation sites on Nter peptide, Figure S2: The prediction results for phosphorylation sites on Cter peptide, Figure S3: The MS/MS chromatogram for goat αs_1_-casein. Table S1: The coverage (%), number of peptides, number of unique peptides, average molecular mass and description of protein identified by PEAKS software.
