# Supplementary material for: Generation of High Affinity Anti-Peptide Polyclonal Antibodies Recognizing Goat αs1-Casein
Source: Molecules. 2020 Jun 5;25(11):2622. doi: 10.3390/molecules25112622 (PMC7321099; doi:10.3390/molecules25112622)
Supplement: Supplementary file 1 [file molecules-25-02622-s001.zip › Supplementary materials.pdf]

Supplementary materials.

NetPhos 3.1 Server - prediction results

Technical University of Denmark

```
>Sequence       18 amino acids
#
# netphos-3.1b prediction results
#
# Sequence                 # x   Context     Score   Kinase    Answer
# -------------------------------------------------------------------
# Sequence                12 S   HRGLSPEVP   0.988   unsp       YES
# Sequence                12 S   HRGLSPEVP   0.515   RSK        YES
# Sequence                12 S   HRGLSPEVP   0.508   GSK3       YES
# Sequence                12 S   HRGLSPEVP   0.466   p38MAPK     .
# Sequence                12 S   HRGLSPEVP   0.461   CaM-II      .
# Sequence                12 S   HRGLSPEVP   0.453   cdk5        .
# Sequence                12 S   HRGLSPEVP   0.441   PKA         .
# Sequence                12 S   HRGLSPEVP   0.416   ATM         .
# Sequence                12 S   HRGLSPEVP   0.408   cdc2        .
# Sequence                12 S   HRGLSPEVP   0.370   CKI         .
# Sequence                12 S   HRGLSPEVP   0.350   DNAPK       .
# Sequence                12 S   HRGLSPEVP   0.300   PKG         .
# Sequence                12 S   HRGLSPEVP   0.284   CKII        .
# Sequence                12 S   HRGLSPEVP   0.257   PKB         .
# Sequence                12 S   HRGLSPEVP   0.091   PKC         .
#
    RPKHPINHRGLSPEVPNE                                #     50
%1  ...........S......
```

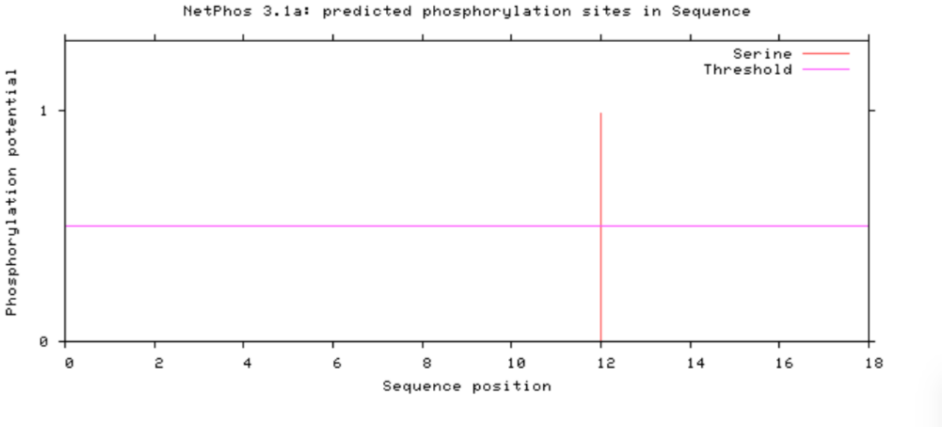

Figure S1: The prediction results for phosphorylation sites on Nter peptide.

NetPhos 3.1 Server - prediction re[illegible]

Technical University of Denmark

```
>Sequence       15 amino acids
#
# netphos-3.1b prediction results
#
# Sequence                  # x   Context     Score   Kinase    Answer
# -------------------------------------------------------------------
# Sequence                  4 S   -PIGSENSG   0.517   CKI        YES
# Sequence                  4 S   -PIGSENSG   0.474   cdc2        .
# Sequence                  4 S   -PIGSENSG   0.445   GSK3        .
# Sequence                  4 S   -PIGSENSG   0.441   DNAPK       .
# Sequence                  4 S   -PIGSENSG   0.439   CaM-II      .
# Sequence                  4 S   -PIGSENSG   0.370   CKII        .
# Sequence                  4 S   -PIGSENSG   0.316   p38MAPK     .
# Sequence                  4 S   -PIGSENSG   0.302   PKG         .
# Sequence                  4 S   -PIGSENSG   0.278   RSK         .
# Sequence                  4 S   -PIGSENSG   0.268   ATM         .
# Sequence                  4 S   -PIGSENSG   0.221   PKC         .
# Sequence                  4 S   -PIGSENSG   0.215   cdk5        .
# Sequence                  4 S   -PIGSENSG   0.192   PKA         .
# Sequence                  4 S   -PIGSENSG   0.092   PKB         .
# Sequence                  4 S   -PIGSENSG   0.005   unsp        .
#
# Sequence                  7 S   GSENSGKTT   0.970   unsp       YES
# Sequence                  7 S   GSENSGKTT   0.526   CKI        YES
# Sequence                  7 S   GSENSGKTT   0.461   CaM-II      .
# Sequence                  7 S   GSENSGKTT   0.447   cdc2        .
# Sequence                  7 S   GSENSGKTT   0.427   GSK3        .
# Sequence                  7 S   GSENSGKTT   0.354   DNAPK       .
# Sequence                  7 S   GSENSGKTT   0.333   CKII        .
# Sequence                  7 S   GSENSGKTT   0.275   ATM         .
# Sequence                  7 S   GSENSGKTT   0.273   p38MAPK     .
# Sequence                  7 S   GSENSGKTT   0.250   RSK         .
# Sequence                  7 S   GSENSGKTT   0.235   PKC         .
# Sequence                  7 S   GSENSGKTT   0.227   PKG         .
# Sequence                  7 S   GSENSGKTT   0.153   PKA         .
# Sequence                  7 S   GSENSGKTT   0.150   cdk5        .
# Sequence                  7 S   GSENSGKTT   0.082   PKB         .
#

# Sequence                 10 T   NSGKTTMPL   0.451   GSK3        .
# Sequence                 10 T   NSGKTTMPL   0.431   CaM-II      .
# Sequence                 10 T   NSGKTTMPL   0.400   unsp        .
# Sequence                 10 T   NSGKTTMPL   0.368   cdc2        .
# Sequence                 10 T   NSGKTTMPL   0.368   CKI         .
# Sequence                 10 T   NSGKTTMPL   0.338   DNAPK       .
# Sequence                 10 T   NSGKTTMPL   0.335   CKII        .
# Sequence                 10 T   NSGKTTMPL   0.333   p38MAPK     .
# Sequence                 10 T   NSGKTTMPL   0.294   RSK         .
# Sequence                 10 T   NSGKTTMPL   0.262   PKG         .
# Sequence                 10 T   NSGKTTMPL   0.255   ATM         .
# Sequence                 10 T   NSGKTTMPL   0.190   cdk5        .
# Sequence                 10 T   NSGKTTMPL   0.189   PKA         .
# Sequence                 10 T   NSGKTTMPL   0.185   PKC         .
# Sequence                 10 T   NSGKTTMPL   0.098   PKB         .
#
# Sequence                 11 T   SGKTTMPLW   0.456   GSK3        .
# Sequence                 11 T   SGKTTMPLW   0.452   CKII        .
# Sequence                 11 T   SGKTTMPLW   0.426   CaM-II      .
# Sequence                 11 T   SGKTTMPLW   0.381   PKG         .

# Sequence                 11 T   SGKTTMPLW   0.379   cdc2        .
# Sequence                 11 T   SGKTTMPLW   0.357   CKI         .
# Sequence                 11 T   SGKTTMPLW   0.349   DNAPK       .
# Sequence                 11 T   SGKTTMPLW   0.345   PKA         .
# Sequence                 11 T   SGKTTMPLW   0.323   p38MAPK     .
# Sequence                 11 T   SGKTTMPLW   0.305   PKC         .
# Sequence                 11 T   SGKTTMPLW   0.279   ATM         .
# Sequence                 11 T   SGKTTMPLW   0.278   RSK         .
# Sequence                 11 T   SGKTTMPLW   0.265   cdk5        .
# Sequence                 11 T   SGKTTMPLW   0.096   PKB         .
# Sequence                 11 T   SGKTTMPLW   0.040   unsp        .
#
    PIGSENSGKTTMPLW                                  #    50
%1  ...S..S........
```

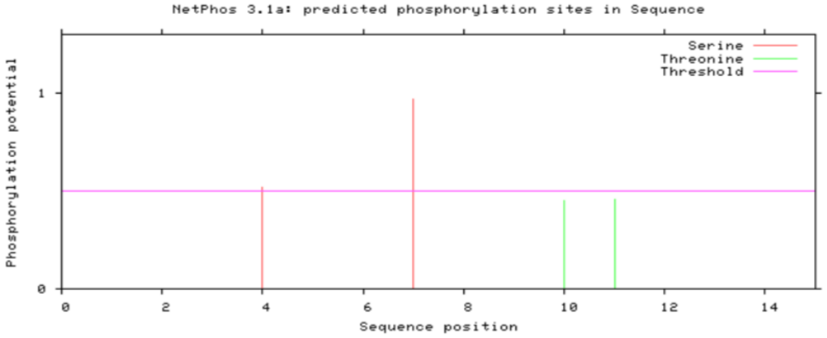

Figure S2: The prediction results for phosphorylation sites on Cter peptide.

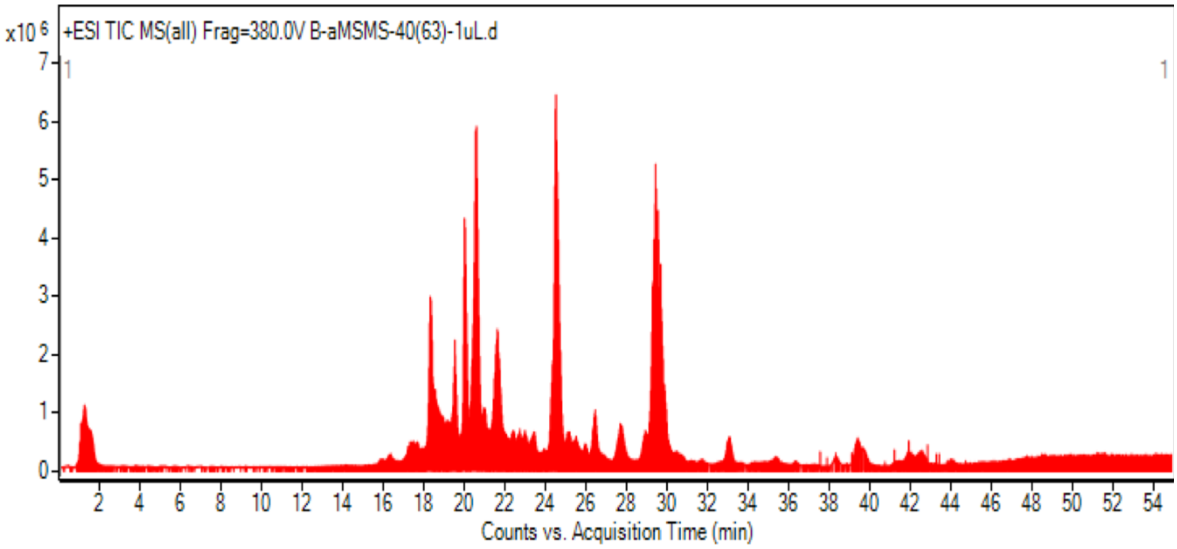

Figure S3. The MS/MS chromatogram for goat α_{S1}-casein.

Table S1. The coverage (%), number of peptides, number of unique peptides, average molecular mass and description of protein identified by PEAKS software.

Accession	Coverage (%)	#Peptides	#Unique	Avg. Mass	Description
P18626|CASA1_CAPHI	79	62	62	24290	Alpha-S1-casein OS=Capra hircus
P33049|CASA2_CAPHI	51	15	15	26389	Alpha-S2-casein OS=Capra hircus
P33048|CASB_CAPHI	39	7	7	24865	Beta-casein OS=Capra hircus
P02670|CASK_CAPHI	21	4	4	21441	Kappa-casein OS=Capra hircus

OS: Organism species
