# Supplementary figures and images for: Generation of High Affinity Anti-Peptide Polyclonal Antibodies Recognizing Goat αs1-Casein
Source: Molecules. 2020 Jun 5;25(11):2622. doi: 10.3390/molecules25112622 (PMC7321099; doi:10.3390/molecules25112622)

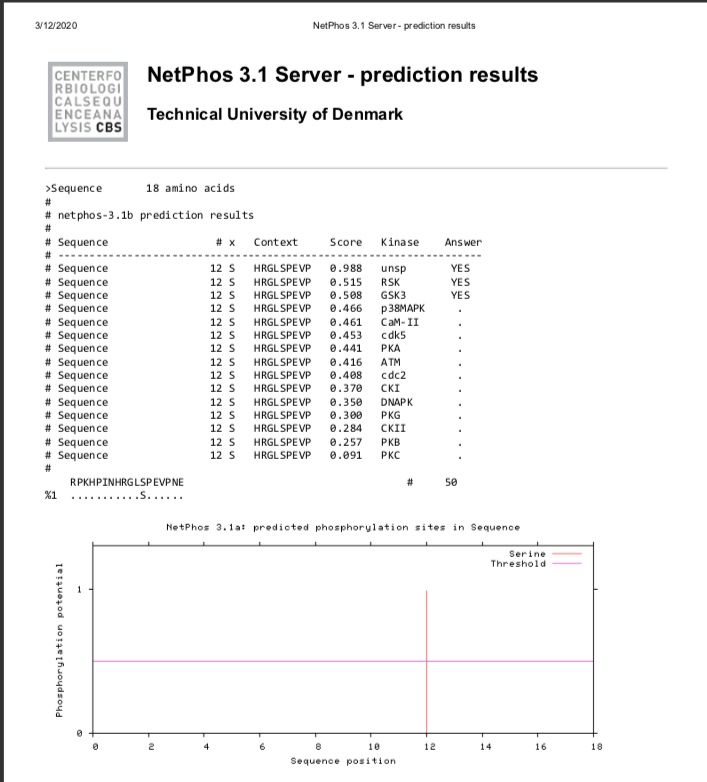

Supplement: Supplementary file 1 [file molecules-25-02622-s001.zip › Figure S1.jpg]

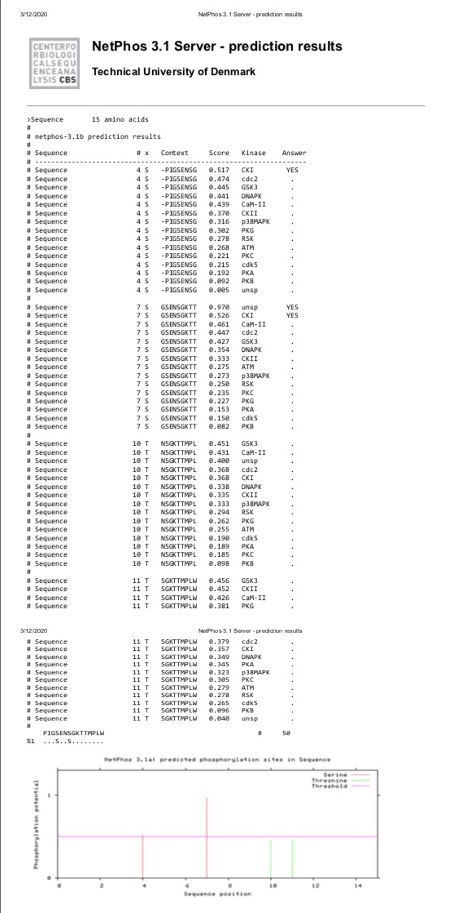

Supplement: Supplementary file 1 [file molecules-25-02622-s001.zip › Figure S2.jpg]

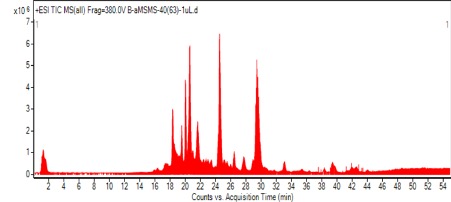

Supplement: Supplementary file 1 [file molecules-25-02622-s001.zip › Figure S3.jpg]
